# Avidity of anti-malarial antibodies inversely related to transmission intensity at three sites in Uganda

**DOI:** 10.1186/s12936-017-1721-3

**Published:** 2017-02-10

**Authors:** Isaac Ssewanyana, Emmanuel Arinaitwe, Joaniter I. Nankabirwa, Adoke Yeka, Richard Sullivan, Moses R. Kamya, Philip J. Rosenthal, Grant Dorsey, Harriet Mayanja-Kizza, Chris Drakeley, Bryan Greenhouse, Kevin K. A. Tetteh

**Affiliations:** 1 Infectious Diseases Research Collaboration, Kampala, Uganda; 2 Department of Medicine, San Francisco General Hospital, University of California, San Francisco, CA USA; 3 Department of Medicine, Makerere University College of Health Sciences, Kampala, Uganda; 4 London School of Hygiene and Tropical Medicine, London, UK

**Keywords:** *Plasmodium falciparum*, Malaria, Antibody, Avidity, Transmission, Uganda

## Abstract

**Background:**

People living in malaria endemic areas acquire protection from severe malaria quickly, but protection from clinical disease and control of parasitaemia is acquired only after many years of repeated infections. Antibodies play a central role in protection from clinical disease; however, protective antibodies are slow to develop. This study sought to investigate the influence of *Plasmodium falciparum* exposure on the acquisition of high-avidity antibodies to *P. falciparum* antigens, which may be associated with protection.

**Methods:**

Cross-sectional surveys were performed in children and adults at three sites in Uganda with varied *P. falciparum* transmission intensity (entomological inoculation rates; 3.8, 26.6, and 125 infectious bites per person per year). Sandwich ELISA was used to measure antibody responses to two *P. falciparum* merozoite surface antigens: merozoite surface protein 1-19 (MSP1-19) and apical membrane antigen 1 (AMA1). In individuals with detectable antibody levels, guanidine hydrochloride (GuHCl) was added to measure the relative avidity of antibody responses by ELISA.

**Results:**

Within a site, there were no significant differences in median antibody levels between the three age groups. Between sites, median antibody levels were generally higher in the higher transmission sites, with differences more apparent for AMA-1 and in ≥5 year group. Similarly, median avidity index (proportion of high avidity antibodies) showed no significant increase with increasing age but was significantly lower at sites of higher transmission amongst participants ≥5 years of age. Using 5 M GuHCl, the median avidity indices in the ≥5 year group at the highest and lowest transmission sites were 19.9 and 26.8, respectively (p = 0.0002) for MSP1-19 and 12.2 and 17.2 (p = 0.0007) for AMA1.

**Conclusion:**

Avidity to two different *P. falciparum* antigens was lower in areas of high transmission intensity compared to areas with lower transmission. Appreciation of the mechanisms behind these findings as well as their clinical consequences will require additional investigation, ideally utilizing longitudinal data and investigation of a broader array of responses.

## Background

Malaria caused by *Plasmodium falciparum* is a major global public health challenge, accounting for an estimated 214 million clinical cases and 438,000 deaths in 2015 [1]. People living in endemic areas acquire protection from the most severe manifestations of malaria relatively quickly, but protection from uncomplicated clinical disease and control of parasitaemia takes longer and is often acquired only after many years of repeated infections [2]. Moreover, sterile immunity to *P. falciparum* is rarely if ever achieved. Passive transfer of immunoglobulin from clinically immune donors to non-immune individuals with *P. falciparum* infection alleviated clinical symptoms and reduced the levels of blood stage parasites, indicating that antibodies play a central role in clinical immunity to malaria [3, 4]. However, numerous studies have measured antibody levels to various *P. falciparum* antigens with conflicting results regarding correlates of protection [5–10]. The qualities of protective antibodies and precise mechanisms by which they mediate protection are not fully understood.

Antibody properties including breadth of response, isotype composition, and avidity appear to play an important role in protective immunity [11–13]. Antibody avidity reflects the overall strength of interaction between the antibody and antigen complex and correlates with protection in naturally acquired and vaccine induced immunity to viral and bacterial pathogens [14–17]. In *P. falciparum* infection, avidity to whole schizont extract, as well as to a number of specific antigens, has been shown to correlate with protection [18–21]. However, acquisition of high antibody avidity to *P. falciparum* antigens with increasing age and varied *P. falciparum* exposure intensity is poorly understood.

In general, repeated exposure to an antigen results in germinal centre reactions which lead to affinity maturation and increases in antibody avidity [22–24]. However, there is also evidence to suggest that *P. falciparum* infection directly interferes with B cell function [2, 8, 25] and disrupts germinal centre architecture [26, 27], potentially interfering with affinity maturation [ [26, 27]. One study performed in a setting of unstable malaria transmission showed evidence of increased antibody avidity following resolution of a clinical malaria episode [18]. In contrast, two studies of children living in endemic areas failed to observe an increase in avidity to a number of *P. falciparum* antigens with increasing exposure [28, 29]. Overall, it is unclear if repeated exposure to *P. falciparum* leads to increased avidity of antibodies directed against plasmodium antigens.

To determine the influence of *P. falciparum* exposure on the natural acquisition of high-antibody avidity, avidity indices to two *P. falciparum* merozoite surface antigens were measured in individuals across a wide range of ages from cross-sectional surveys performed in three sites in Uganda with varying transmission intensity. Antibody avidity indices were then compared between ages and sites to determine whether there were differences in antibody avidity associated with age and *P. falciparum* exposure intensity.

## Methods

### Study sites and cross-sectional surveys

This study took place in three sub-counties in Uganda with varied *P. falciparum* transmission intensity. Walukuba, a peri-urban area near Lake Victoria, had relatively low transmission intensity, with an entomological inoculation rate (EIR) estimated at 3.8 infectious bites per person per year (IBPPY) [30]. Kihihi, a rural area in the south-western part of Uganda, had higher transmission, with an estimated EIR of 26.6 IBPPY. Nagongera, a rural area in the south-eastern part of the country, had the highest transmission, with an estimated EIR of 125 IBPPY. Malaria transmission at all three sites was perennial. Malaria control interventions in all three districts included use of long lasting insecticide treated nets (LLIN), malaria case management with artemisinin based therapies, and intermittent presumptive treatment during pregnancy with sulfadoxine–pyrimethamine.

Cross-sectional surveys were conducted between January and June 2012 at all three sites [31]. Survey staff recruited members from 200 households randomly selected from a population-based census. Dried blood spots (DBS) from finger-prick samples were obtained from all children under 15 years of age and from a random selection of age stratified adults in the following categories: 15–24, 25–34, 35–44, 45–54, and >55 years. The cross-sectional survey resulted in recruitment of 2737 participants of which 2227 had ELISA results. For this study, 581 and 1029 participants who had reactive antibodies with normalized OD >0.5 were analysed for avidity to MSP-1 and AMA-1 respectively. This allowed inclusion of participants who might have had a more recent antibody boost for a more direct comparison across sites.

### Ethical approval and informed consent

Ethical approval was obtained from the Makerere University School of Medicine Research and Ethics Committee (REC REF 2011–203), Uganda National Council for Science and Technology (HS 1074), London School of Hygiene & Tropical Medicine Ethics Committee (Reference 6012), and the University of California, San Francisco Committee on Human Research (Reference 027911). Written informed consent was obtained from the parents/guardians on behalf of the children enrolled in the study and from all participants above 18 years of age. Assent was obtained from all children 8–17 years.

### Elution of antibodies from dried blood spots (DBS)

DBS were collected on Whatman 3MM filter paper and stored at −20 °C. For this study, a 3 mm diameter punch of DBS was hydrated in 200 µl of phosphate buffered saline (PBS) containing 0.005% Tween 20 and 0.01% sodium azide. The samples were left on a plate shaker overnight at room temperature before storage at 4 °C. The excised spot was estimated to contain approximately 2 µl plasma, resulting in a serum dilution factor of 1:200 [32].

### Modified ELISA to measure the avidity index

Antibodies to MSP1-19 (Wellcome strain) [33] and AMA-1 (FVO strain) [34] were measured via a standard sandwich ELISA method [28, 31]. In brief, Immulon-4 HBX microtitre plates (Thermo Labsystems, Basingstoke, UK) were coated overnight at 4 °C with 0.5 mg/ml of antigen in coating buffer (0.1 M sodium carbonate/bicarbonate, pH 9.6), and then washed three times with wash buffer; PBS, 0.05% Tween-20. Plates were blocked for 3 h at room temperature with 200 µl/well PBS, 0.05% Tween-20, 1% skimmed dried milk and then washed three times. For each sample, plasma was diluted to a final concentration of 1:1000 for MSP1-19 and 1:2000 for AMA1, with 50 μl of diluted plasma added per well. Plates were incubated overnight at 4 °C and washed six times. HRP-conjugated rabbit anti-human IgG (Dako Ltd, High Wycombe, UK) was diluted 1:5000 in blocking buffer and 50 μl was added per well. Plates were incubated for 3 h at room temperature, then washed six times and developed with 100 µl/well of *o*-phenylenediamine (OPD)–H_2_O_2_. The reaction was stopped after 15 min with 25 μl 2 M sulphuric acid. ODs were measured at 492 nm using a VERSAmax plate reader, with Softmax software (Molecular Devices, USA).

To evaluate antibody avidity, the sandwich ELISA assay described above was modified to include an antibody disassociation step prior to addition of HRP-conjugated secondary antibody [29, 35, 36]. Briefly, diluted plasma samples were incubated on the plates overnight at 4 °C, antibody avidity was measured by treating duplicate wells with 2 or 5 M guanidine hydrochloride (GuHCl) for 10 min and then washing six times. PBS was used for the control wells to measure the total antibody binding. Test samples with and without GuHCl were run on the same plate to minimize variability. The avidity index (AI) was defined as the proportion of antibodies binding after treatment with GuHCl for each dilution (Avidity index = [OD following GuHCl treatment/OD without GuHCl treatment] × 100).

In order to overcome the limitations of avidity measurement by ELISA (poor resolution at the lower OD), and to select for participants with a relatively recent antibody boost across the three sites, only samples with titers above a normalized OD (adjusted OD based on a positive control to correct for inter-plate variations) of 0.5 were included in the assessment of the avidity index. In each plate, hyper-immune sera was included at a dilution 1:3200 as a positive control.

### Data analysis

Participants were stratified into age groups 1–4, 5–15 and >15 years. Non-parametric comparison of medians across groups within a site and across sites for the different age groups was performed using the Mann–Whitney test. Correlations were performed using the Pearson test. Graphs were plotted using Graphpad PRISM version 5 (Graphpad Software, USA).

## Results

### Antibody levels in three sites in Uganda

Antibody levels and avidity index (AI) were measured in samples from cross sectional surveys conducted at three sites in Uganda. Consistent with published data on antibody prevalence [31], the proportion of all cross-sectional survey samples included in this study (those with a normalized OD ≥ 0.5) increased with transmission intensity for AMA-1 (29.1, 43.6, and 67.7% for Walukuba, Kihihi and Nagongera, respectively) but not MSP1-19 (14.6, 34.5, and 27.3% for Walukuba, Kihihi and Nagongera, respectively). Amongst included samples, there were no significant differences in median antibody levels between the three age groups (1–4, 5–15 and >15 years) for either MSP1-19 or AMA-1 within each of the three sites. However, median antibody levels were generally higher in the higher transmission sites, with differences more apparent for AMA-1 and in older individuals (Table 1).

**Table 1:** Antibody responses to MSP1-19 and AMA-1

Antigen	Age group (years)	Site	No of participants	Median age (IQR)	Median OD (IQR)	p value		
						Walukuba vs Kihihi	Walukuba vs Nagongera	Kihihi vs Nagongera
MSP1-19	1–4	Walukuba	12	3 (2–4)	0.71 (0.60–1.10)	0.08	*0.041*	0.44
		Kihihi	18	3 (1–4)	0.90 (0.70–1.17)			
		Nagongera	24	1 (1–3)	1.00 (0.73–1.29)			
	5–15	Walukuba	16	12 (6–13)	0.80 (0.65–1.25)	0.5	0.4	0.8
		Kihihi	76	12 (8–13)	0.89 (0.63–1.12)			
		Nagongera	74	11 (7–13)	0.87 (0.63–1.17)			
	>15	Walukuba	64	28 (20–40)	0.82 (0.61–1.1)	*0.029*	*0.011*	0.4
		Kihihi	181	35 (25–48)	0.97 (0.70–1.27)			
		Nagongera	116	39 (25–55)	1.00 (0.67–1.30)			
Total			581					
AMA-1	1–4	Walukuba	18	2 (1–3)	0.86 (0.59–1.10)	0.95	0.18	0.19
		Kihihi	21	3 (2–4)	0.79 (0.65–1.11)			
		Nagongera	63	3 (2–4)	0.92 (0.67–1.2)			
	5–15	Walukuba	76	9 (6–12)	0.92 (0.67–1.07)	*0.005*	*0.0001*	0.6
		Kihihi	125	11 (8–13)	1.1 (0.80–1.15)			
		Nagongera	251	9 (7–12)	1.1 (0.85–1.2)			
	>15	Walukuba	89	27 (22–32)	0.72 (0.61–0.88)	*0.0001*	*0.0007*	0.62
		Kihihi	197	34 (22–47)	0.85 (0.68–1.1)			
		Nagongera	189	34 (22–50)	0.87 (0.65–1.1)			
Total			1029					

Statistically significant values (p < 0.05) are in italics

### Relationship between age and antibody avidity

**Fig. 1:**
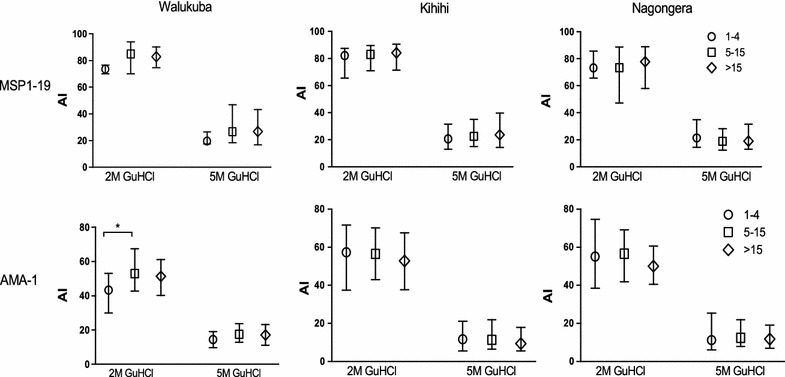
Avidity index to MSP1-19 and AMA-1 across age groups within the three sites, at the two GuHCl concentrations (2 and 5 M). The *bars* represent the median and interquartile range. *p < 0.05

Median avidity index to MSP1-19 at 2 and 5 M GuHCl was not significantly different between age groups at all three sites. Similarly, median avidity index to AMA-1 at 2 and 5 M GuHCl showed no difference between age groups at all three sites, with the exception of 1–4 versus 5–15 year olds in Walukuba (73.6 versus 85.1 p = 0.04) (Fig. 1).

### Relationship between malaria transmission and antibody avidity

**Fig. 2:**
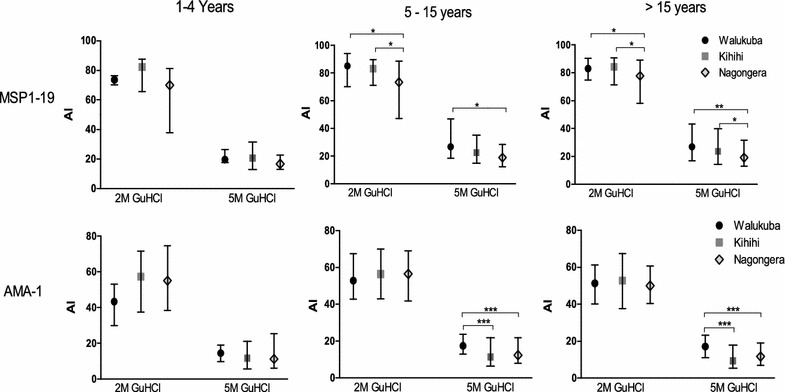
Avidity index to MSP1-19 and AMA-1 across transmission sites within the three age groups, at the two GuHCl concentrations (2 and 5 M). The *bars* represent the median and interquartile range. *p < 0.05, **p < 0.001, ***p < 0.0001

In contrast to the trends observed in antibody levels, the avidity index to MSP1-19 was significantly lower in the highest malaria transmission site of Nagongera than Kihihi and Walukuba in those over five, at both GuHCl concentrations (2 and 5 M) (Fig. 2). There were no significant differences in avidity index between sites in children under five. Responses to AMA-1 showed a similar pattern, with avidity index lower in Nagongera and Kihihi than Walukuba in those at least 5 years old at 5 M. There was no evidence of correlation between the avidity index to MSP1-19 and AMA-1 at either GuHCl concentration (r^2^ < 0.002, p > 0.5 at 2 and 5 M GuHCl, for all three sites). Overall, these findings suggest an inverse relationship between avidity index and both transmission intensity and antibody response to MSP1-19 and AMA-1 across sites for those above 5 years.

## Discussion

This study sought to determine the influence of *P. falciparum* exposure on natural acquisition of high avidity antibodies. Avidity was measured to MSP1-19 and AMA-1 antigens in 581 and 1029 individuals respectively encompassing a wide range of ages, from three sites in Uganda with transmission intensities ranging from moderate to extremely high. These results demonstrated that age had a minimal effect on antibody avidity, consistent with previous studies using similar methods of evaluation, which observed slight or inconsistent age-related differences in avidity to MSP1-19 and AMA-1 [28, 29]. Importantly, despite higher antibody levels, avidity to both antigens was significantly lower at the site of highest *P. falciparum* transmission intensity in children ≥5 years and adults. These results suggest that affinity maturation to *P. falciparum* antigens may be compromised in the setting of very high, perennial exposure to this parasite.

There are at least two potential explanations for this study’s main findings, which are neither exhaustive nor mutually exclusive: (1) near constant exposure to antigen may impair maturation/persistence of avid antibodies via a number of potential mechanisms; and (2) recent infection may result in addition of low avid antibodies to the circulation, reducing the proportion which have high avidity.

Affinity maturation is acquired through somatic hypermutation during germinal centre reactions, with B cell affinity to antigen driving selection that results in the production of higher affinity antibodies [37, 38]. Classical understanding of B cell biology would suggest that repeated exposure would be expected to drive several rounds of germinal centre reactions, ultimately resulting in higher antibody affinities to the pathogen, hence higher antibody avidity. In contrast, the results showed a lower proportion of avid antibodies in participants living in the highest *P. falciparum* transmission sites, where exposure to antigen is most frequent. Animal studies suggest that germinal centre development is temporally ordered, with short lived plasma cells (SLPC) emerging from germinal centres initially, followed by memory B cells (MBC) and then, weeks to months later, long lived plasma cells (LLPC) that have undergone extensive affinity maturation [37, 39]. Disturbance to this process resulting from frequent or chronic infections may compromise the ultimate development of high affinity MBC and LLPC. For example, there is evidence that frequent acute malaria interferes with follicular helper T cell differentiation and germinal centre architecture in the spleen [27, 40–42]. Another hypothesis suggests that *P. falciparum* infection is associated with dysfunction of the B cell compartment, including accumulation of atypical memory B cells [43], which could theoretically result in the inefficient acquisition and maintenance of antibody mediated immunity to malaria [44]. It is also possible that frequent exposure to a large number of antigen clones may impair affinity maturation to any one of the clones and it is likely that the highest transmission site experience a higher complexity of infection.

In the presence of low antigen levels, affinity matured plasma cells that secrete high affinity antibodies should be favored over lower affinity plasma cells and circulating antibodies for survival. These affinity matured plasma cells would therefore maintain the blood antibody levels. However, in the presence of frequent re-infection, a relatively large antigen load may be more likely to promote survival of poorly affinity-matured plasma cells, including T cell-independent or extra-follicular B cell reactions. Furthermore, acute *P. falciparum* infection is associated with non-specific, polyclonal B cell activation that may result in the expansion of low avidity, short lived plasma cells [25]. These factors may reduce the proportion but not necessarily the titers of high avidity antibodies. In contrast, in the absence of recent infection the antibody pool is predominantly comprised of high avidity antibodies generated by LLPC after the contraction of the SLPC. This may explain why Ugandans in lower transmission areas, who on average had a longer duration of time since their last infection, had higher proportions of highly avid antibodies. Based on this argument, most likely the difference in avidity index between the low and high transmission sites should have been more pronounced if the method allowed to examine samples below with OD <0.5.

The impact of the differences in avidity index observed in this study on acquisition of clinical or anti-parasite immunity is not known. While antibody avidity is theoretically important in the function of an antibody response [45], there are few available empiric data to support the relationship between avidity and immunity in malaria. Reddy et al. found that individuals with higher antibody avidity to *P. falciparum* antigens were less likely to experience malaria during follow-up [21, 46]. In an RTS,S malaria vaccine trial, investigators did not find an association between antibody avidity to circumsporozoite protein and protection after the last dose of vaccination among children, but they did show that the change in avidity between second and third vaccination was associated with a 54% reduced risk of acquiring malaria [47, 48]. The relationship between avidity measurements and clinical immunity is also complex, with numerous factors other than avidity at play. High antibody titres may compensate for low avidity; therefore, relatively low avidity in the presence of high antibody levels may not have a negative consequence on naturally acquired immunity. Clinical data from this study show that acquired immunity clearly develops in children in Nagongera as well as the other sites, the study site with highest *P. falciparum* transmission, based on reductions in the incidence of disease with age [49]. However it is not clear whether acquired immunity would develop and be maintained more effectively by decreasing the nearly continuous exposure to blood stage parasites at the site, e.g. via chemoprevention or by reducing transmission using vector control [50].

The cross-sectional nature of this study limited the ability to investigate the mechanisms behind the acquisition of antibody avidity. Furthermore, this study only investigated responses to two of many *P. falciparum* proteins, and it is not yet well established which combinations of responses are most relevant for acquisition of immunity. In addition, while providing an overall estimate of the proportion of the polyclonal antibody pool which is highly avid, the ELISA-based methods used in this study were not able to measure avidity in participants with OD <0.5 or further dissect binding characteristics of the antibodies.

## Conclusion

In conclusion, this study showed that avidity to two different *P. falciparum* antigens was lower in areas of high versus low transmission intensity. The mechanisms behind these findings as well as their clinical consequences, if any, are not yet clear. A more detailed investigation, ideally linked to longitudinal investigation of *P. falciparum* exposure as well as a more comprehensive analysis of the B cell and antibody response, will be valuable in helping to further illuminate these findings.

## References

[1] WHO. World malaria report 2015. Geneva: World Health Organization; 2015. http://www.who.int/malaria/publications/world-malaria-report-2015/report/en/. Accessed 12 Mar 2016.

[2] Langhorne J, Ndungu FM, Sponaas AM, Marsh K (2008). Immunity to malaria: more questions than answers. Nat Immunol.

[3] Cohen S, McGregor IA, Carrington S (1961). Gamma-globulin and acquired immunity to human malaria. Nature.

[4] McGregor IA, Carrington S, Cohen S (1963). Treatment of East African *P. falciparum* malaria with West African human γ-globulin. Trans R Soc Trop Med Hyg.

[5] Druilhe P, Khusmith S (1987). Epidemiological correlation between levels of antibodies promoting merozoite phagocytosis of *Plasmodium falciparum* and malaria-immune status. Infect Immun.

[6] John CC, Moormann AM, Pregibon DC, Sumba PO, McHugh MM, Narum DL (2005). Correlation of high levels of antibodies to multiple pre-erythrocytic *Plasmodium falciparum* antigens and protection from infection. Am J Trop Med Hyg.

[7] Fowkes FJI, Richards JS, Simpson JA, Beeson JG (2010). The relationship between anti-merozoite antibodies and incidence of *Plasmodium falciparum* malaria: a systematic review and meta-analysis. PLoS Med.

[8] Portugal S, Pierce SK, Crompton PD (2013). Young lives lost as B cells falter: what we are learning about antibody responses in malaria. J Immunol.

[9] Tetteh KK, Osier FH, Salanti A, Kamuyu G, Drought L, Failly M (2013). Analysis of antibodies to newly described *Plasmodium falciparum* merozoite antigens supports MSPDBL2 as a predicted target of naturally acquired immunity. Infect Immun.

[10] Crompton PD, Kayala MA, Traore B, Kayentao K, Ongoiba A, Weiss GE (2010). A prospective analysis of the Ab response to *Plasmodium falciparum* before and after a malaria season by protein microarray. Proc Natl Acad Sci USA.

[11] Nogaro SI, Hafalla JC, Walther B, Remarque EJ, Tetteh KK, Conway DJ (2011). The breadth, but not the magnitude, of circulating memory B cell responses to *P. falciparum* increases with age/exposure in an area of low transmission. PLoS ONE.

[12] Groux H, Gysin J (1990). Opsonization as an effector mechanism in human protection against asexual blood stages of *Plasmodium falciparum*: functional role of IgG subclasses. Res Immunol.

[13] Hill DL, Eriksson EM, Li Wai Suen CS, Chiu CY, Ryg-Cornejo V, Robinson LJ (2013). Opsonising antibodies to *P. falciparum* merozoites associated with immunity to clinical malaria. PLoS ONE.

[14] Schlesinger Y, Granoff DM (1992). Avidity and bactericidal activity of antibody elicited by different *Haemophilus influenzae* type b conjugate vaccines. JAMA.

[15] Goldblatt D, Vaz AR, Miller E (1998). Antibody avidity as a surrogate marker of successful priming by *Haemophilus influenzae* type b conjugate vaccines following infant immunization. J Infect Dis.

[16] Bachmann MF, Kalinke U, Althage A, Freer G, Burkhart C, Roost H (1997). The role of antibody concentration and avidity in antiviral protection. Science.

[17] Maynard JA, Maassen CB, Leppla SH, Brasky K, Patterson JL, Iverson BL (2002). Protection against anthrax toxin by recombinant antibody fragments correlates with antigen affinity. Nat Biotechnol.

[18] Ferreira MU, Kimura EAS, de Souza JM, Katzin AM (1996). The isotype composition and avidity of naturally acquired anti-*Plasmodium falciparum* antibodies: differential patterns in clinically immune Africans and Amazonian patients. Am J Trop Med Hyg.

[19] Leoratti FM, Durlacher RR, Lacerda MV, Alecrim MG, Ferreira AW, Sanchez MC (2008). Pattern of humoral immune response to *Plasmodium falciparum* blood stages in individuals presenting different clinical expressions of malaria. Malar J.

[20] Tutterrow YL, Salanti A, Avril M, Smith JD, Pagano IS, Ako S (2012). High avidity antibodies to full-length VAR2CSA correlate with absence of placental malaria. PLoS ONE.

[21] Reddy SB, Anders RF, Beeson JG, Färnert A, Kironde F, Berenzon SK (2012). High affinity antibodies to *Plasmodium falciparum* merozoite antigens are associated with protection from malaria. PLoS ONE.

[22] MacLennan ICM (1994). Germinal centers. Annu Rev Immunol.

[23] Liu Y-J, Arpin C (1997). Germinal center development. Immunol Rev.

[24] Gatto D, Brink R (2010). The germinal center reaction. J Allergy Clin Immunol.

[25] Donati D, Mok B, Chêne A, Xu H, Thangarajh M, Glas R (2006). Increased B cell survival and preferential activation of the memory compartment by a malaria polyclonal B cell activator. J Immunol.

[26] Carvalho LJ, Ferreira-da-Cruz MF, Daniel-Ribeiro CT, Pelajo-Machado M, Lenzi HL (2007). Germinal center architecture disturbance during *Plasmodium berghei* ANKA infection in CBA mice. Malar J.

[27] Alves FA, Pelajo-Machado M, Totino PR, Souza MT, Gonçalves EC, Schneider MP (2015). Splenic architecture disruption and parasite-induced splenocyte activation and anergy in *Plasmodium falciparum*-infected *Saimiri sciureus* monkeys. Malar J.

[28] Akpogheneta OJ, Dunyo S, Pinder M, Conway DJ (2010). Boosting antibody responses to *Plasmodium falciparu*m merozoite antigens in children with highly seasonal exposure to infection. Parasite Immunol.

[29] Ibison F, Olotu A, Muema DM, Mwacharo J, Ohuma E, Kimani D (2012). Lack of avidity maturation of merozoite antigen-specific antibodies with increasing exposure to *Plasmodium falciparum* amongst children and adults exposed to endemic malaria in Kenya. PLoS ONE.

[30] Kilama M, Smith DL, Hutchinson R, Kigozi R, Yeka A, Lavoy G (2014). Estimating the annual entomological inoculation rate for *Plasmodium falciparum* transmitted by *Anopheles gambiae s.l.* using three sampling methods in three sites in Uganda. Malar J.

[31] Yeka A, Nankabirwa J, Mpimbaza A, Kigozi R, Arinaitwe E, Drakeley C (2015). Factors associated with malaria parasitemia, anemia and serological responses in a spectrum of epidemiological settings in Uganda. PLoS ONE.

[32] Corran PH, Cook J, Lynch C, Leendertse H, Manjurano A, Griffin J (2008). Dried blood spots as a source of anti-malarial antibodies for epidemiological studies. Malar J.

[33] Burghaus PA, Holder AA (1994). Expression of the 19-kilodalton carboxy-terminal fragment of the *Plasmodium falciparum* merozoite surface protein-1 in *Escherichia coli* as a correctly folded protein. Mol Biochem Parasitol.

[34] Kocken CH, Withers-Martinez C, Dubbeld MA, van der Wel A, Hackett F, Valderrama A (2002). High-level expression of the malaria blood-stage vaccine candidate *Plasmodium falciparum* apical membrane antigen 1 and induction of antibodies that inhibit erythrocyte invasion. Infect Immun.

[35] Pour Abolghasem S, Bonyadi MR, Babaloo Z, Porhasan A, Nagili B, Gardashkhani OA (2011). IgG avidity test for the diagnosis of acute *Toxoplasma gondii* infection in early pregnancy. Iran J Immunol.

[36] Zakeri S, Babaeekhou L, Mehrizi AA, Abbasi M, Djadid ND (2011). Antibody responses and avidity of naturally acquired anti-*Plasmodium vivax* Duffy binding protein (PvDBP) antibodies in individuals from an area with unstable malaria transmission. Am J Trop Med Hyg.

[37] Shlomchik MJ, Weisel F (2012). Germinal center selection and the development of memory B and plasma cells. Immunol Rev.

[38] Liu Y-J, Joshua DE, Williams GT, Smith CA, Gordon J, MacLennan IC (1989). Mechanism of antigen-driven selection in germinal centres. Nature.

[39] Weisel FJ, Zuccarino-Catania GV, Chikina M, Shlomchik MJ (2016). A temporal switch in the germinal center determines differential output of memory B and plasma cells. Immunity.

[40] Cadman ET, Abdallah AY, Voisine C, Sponaas AM, Corran P, Lamb T (2008). Alterations of splenic architecture in malaria are induced independently of Toll-like receptors 2, 4, and 9 or MyD88 and may affect antibody affinity. Infect Immun.

[41] Ryg-Cornejo V, Ioannidis LJ, Ly A, Chiu CY, Tellier J, Hill DL (2016). Severe malaria infections impair germinal center responses by inhibiting T follicular helper cell differentiation. Cell Rep.

[42] Obeng-Adjei N, Portugal S, Tran TM, Yazew TB, Skinner J, Li S (2015). Circulating Th1 cell-type Tfh cells that exhibit impaired B cell help are preferentially activated during acute malaria in children. Cell Rep.

[43] Weiss GE, Crompton PD, Li S, Walsh LA, Moir S, Traore B (2009). Atypical memory B cells are greatly expanded in individuals living in a malaria-endemic area. J Immunol.

[44] Scholzen A, Sauerwein RW (2013). How malaria modulates memory: activation and dysregulation of B cells in Plasmodium infection. Trends Parasitol.

[45] Cremers AJ, Lut J, Hermans PW, Meis JF, de Jonge MI, Ferwerda G (2014). Avidity of antibodies against infecting pneumococcal serotypes increases with age and severity of disease. Clin Vaccine Immunol.

[46] Reddy SB, Anders RF, Cross N, Mueller I, Senn N, Stanisic DI (2015). Differences in affinity of monoclonal and naturally acquired polyclonal antibodies against *Plasmodium falciparum* merozoite antigens. BMC Microbiol.

[47] Ajua A, Lell B, Agnandji ST, Asante KP, Owusu-Agyei S, Mwangoka G (2015). The effect of immunization schedule with the malaria vaccine candidate RTS, S/AS01E on protective efficacy and anti-circumsporozoite protein antibody avidity in African infants. Malar J.

[48] Olotu A, Clement F, Jongert E, Vekemans J, Njuguna P, Ndungu FM (2014). Avidity of anti-circumsporozoite antibodies following vaccination with RTS, S/AS01E in young children. PLoS ONE.

[49] Kamya MR, Arinaitwe E, Wanzira H, Katureebe A, Barusya C, Kigozi SP (2015). Malaria transmission, infection, and disease at three sites with varied transmission intensity in Uganda: implications for malaria control. Am J Trop Med Hyg.

[50] Jagannathan P, Bowen K, Nankya F, McIntyre TI, Auma A, Wamala S (2016). Effective antimalarial chemoprevention in childhood enhances the quality of CD4+ T cells and limits their production of immunoregulatory interleukin 10. J Infect Dis.

